# A New Algorithm for Integrated Analysis of miRNA-mRNA Interactions Based on Individual Classification Reveals Insights into Bladder Cancer

**DOI:** 10.1371/journal.pone.0064543

**Published:** 2013-05-24

**Authors:** Nikolai Hecker, Carsten Stephan, Hans-Joachim Mollenkopf, Klaus Jung, Robert Preissner, Hellmuth-A. Meyer

**Affiliations:** 1 Center for Bioinformatics, University of Hamburg, Hamburg, Germany; 2 Institute of Physiology, Charité - Universitätsmedizin Berlin, Berlin, Germany; 3 Department of Urology, Charité - Universitätsmedizin Berlin, Berlin, Germany; 4 Berlin Institute for Urologic Research, Berlin, Germany; 5 Core Facility Genomics/Microarray, Max Planck Institute for Infection Biology, Berlin, Germany; University of Pittsburgh, United States of America

## Abstract

**Background:**

MicroRNAs (miRNAs) are small non-coding RNAs that regulate gene expression. It has been proposed that miRNAs play an important role in cancer development and progression. Their ability to affect multiple gene pathways by targeting various mRNAs makes them an interesting class of regulators.

**Methodology/Principal Findings:**

We have developed an algorithm, Classification based Analysis of Paired Expression data of RNA (CAPE RNA), which is capable of identifying altered miRNA-mRNA regulation between tissues samples that assigns interaction states to each sample without preexisting stratification of groups. The distribution of the assigned interaction states compared to given experimental groups is used to assess the quality of a predicted interaction. We demonstrate the applicability of our approach by analyzing urothelial carcinoma and normal bladder tissue samples derived from 24 patients. Using our approach, normal and tumor tissue samples as well as different stages of tumor progression were successfully stratified. Also, our results suggest interesting differentially regulated miRNA-mRNA interactions associated with bladder tumor progression.

**Conclusions/Significance:**

The need for tools that allow an integrative analysis of microRNA and mRNA expression data has been addressed. With this study, we provide an algorithm that emphasizes on the distribution of samples to rank differentially regulated miRNA-mRNA interactions. This is a new point of view compared to current approaches. From bootstrapping analysis, our ranking yields features that build strong classifiers. Further analysis reveals genes identified as differentially regulated by miRNAs to be enriched in cancer pathways, thus suggesting biologically interesting interactions.

## Introduction

Bladder cancer is the fourth most common cancer in industrialized countries [1]. Muscle invasive bladder carcinoma has still a high mortality, despite better therapies by improved surgical techniques and aggressive treatments. Approximately 90% of all urothelial neoplasms are classified as urothelial cell carcinoma (UCC), which can be divided by clinical and morphologic parameters in two different subgroups [2], [3]. The majority of UCC belongs to the group of papillary non-invasive tumors (stage pTa), in general these tumors are well differentiated, tend to grow slowly without large spreading and have a good clinical prognosis. The remaining one-third of UCC are invasive tumors (stage pT1 and higher) with poorly differentiation, high progression rates and the ability to form metastases. On the molecular level, most non-invasive UCC are associated with FGFR3 mutation and chromosome 9 loss [4], [5] whereas the inactivation of p53 and PTEN function plays an important role in the progression of invasive UCC [6]. In several publications, transcriptomic expression patterns have been linked to clinical outcomes in urothelial carcinoma [7]–[10]. Furthermore, first integrated analysis of both miRNA and mRNA data was performed to get a more detailed insight into regulatory networks and involved cancer signal transduction pathways that cause bladder cancer [11], [12]. However, the exact mechanisms involved in the initiation and progression of bladder urothelial carcinoma remain largely unclear. Further examination of gene expression and miRNA expression data is crucial to detect those unknown processes that lead to tumorgenesis. With the establishment of microarray applications, several computational methods have been developed to analyze gene expression data. Gene set analysis and gene enrichment analysis are often used to identify differentially expressed genes [13], [14]. The most common tools and web services that apply the principles of gene enrichment analysis are DAVID [15], GeneTrail [16], GOrilla [17], GeneCodis [18] and GOEAST [19], for a general overview see reference [20].

Apart from co-expressed genes, differentially regulated pairs of miRNAs and mRNAs play an important role in several cellular processes and diseases. To assess this issue, several methods have been developed to predict interactions between miRNAs and mRNAs based on their sequences. Most of the tools exploit the seed complementary between miRNAs and the 3′UTR of specific mRNA, information about the sequence conservation of adjacent bases and thermodynamic properties of miRNA-target mRNA interactions. The different methods have been recently reviewed [21]. Some of the most common tools are TargetScan [22]–[25], PicTar [26]–[29], miRanda [30]–[32] and PITA [33]. Several web resources provide validated or predicted miRNA-mRNA interactions, e.g. TarBase [34], miRecords [35], miRGen [36] and miRBase [37], miRGator offers miRNA and mRNA expression profiles [38], starBase [39] and doRiNA [40] are databases that integrate miRNA and ribonucleoprotein binding sites.

There is a need for methods which consider the specific nature of miRNA induced regulation. miReduce [41] and Sylamer [42] can be used to evaluate the correlation between seed motif enrichments in 3′UTRs of mRNAs for differentially regulated genes in miRNA knockout experiments. DIANA-mirExTra implements similar gene motif evaluation methods as a web service [43]. Creighton et al developed a collection of Excel macros to combine sets of enriched genes with miRNA-mRNA interaction predictions [44]. Recently, methods and web-services for the integrated analysis of miRNA and mRNA expression data have been developed such as MAGIA [45], [46], MMIA [47], mirAct [48], miRConnX [49] and miRTrail [50]. GenMIR++ implements a Bayesian learning approach to identify differential miRNA-mRNA regulation [51], [52]. HOCTAR calculates negative correlations between miRNA and mRNA expression [53]. Other methods are based on regression analysis [54], [55]. An approach based on clustering miRNA and mRNA expression data in combination with a t-test was developed by Jayaswal et al. [56]. Most of current tools have shortcomings such as using methods that are error-prone to outliers or they do not allow identifying differential regulation between two groups of samples.

In this study, we present a novel approach that evaluates differential miRNA-mRNA regulation combined with the distribution of samples for a single interaction. We hypothesize that single miRNA-mRNA interactions are characteristic for a particular state of tumorigenesis. We consider differential miRNA induced gene regulation as a two class problem and use the following assumption. Given an interaction between a miRNA and mRNA which is characteristic for a difference between two groups of samples, the miRNA is up-regulated and the mRNA down-regulated in the first group with respect to the second group, or reciprocal. Our approach classifies each predicted interaction for each sample independently of group knowledge. By this way, one can analyze individual differences inside a collective of samples for a specific set of interactions. Furthermore given an interaction, we can partition samples into expected groups which reflect the miRNA induced gene regulation. The agreement between the expected groups and the experimental ones yields a meaningful ranking to distinguish potential interactions from those which are unlikely to occur. In a final step, we incorporate information about negative correlation between miRNA and mRNA expression to eliminate false positives.

Identifying differentially regulated miRNA-mRNA interactions is a basically a form of feature selection. To validate the different steps of our approach, we have performed a principal component analysis to analyze the separation of samples after assignment of interaction states and evaluated the performance of our ranking to build classifiers.

In particular, we have applied our approach to a collective of healthy bladder tissue samples and bladder tumor samples at different stages. In addition, we have examined the ability of our approach to classify prostate cancer tumors and healthy tissue, as well as colon cancer samples and healthy tissue using small sample sizes [57]. The performance of our classifiers was compared to a well established method for gene expression data, Prediction Analysis of Microrarrays for R (pamr), which is an enhanced nearest centroid classifier [58]. Furthermore, we calculated pathway enrichment scores for genes involved in predicted interactions and suggest interesting interactions for bladder cancer tumor progression.

## Materials and Methods

### Patients and Tissue Samples

A selection of 24 urothelial samples from a collective of bladder cancer patients described previously was used in this study [59]. Eight samples were extracted from nonmalignant bladder tissue (8 male patients; median age 69, range 47–80 years), 8 samples from low-grade papillary urothelial carcinoma (8 male patients; median age 72.5, range 59–79 years; 2x pTaG1 and 6x pTaG2)), and 8 samples from invasive tumors (6 male, 2 female patients; median age 73, range 62–76 years; 1x pT1G1, 4x pT1G3 and 3x pT2G3). The samples were collected immediately after surgery in liquid nitrogen and stored at −80°C until further analysis. Tumor staging was performed in conformity with the International Union Against Cancer and histological grading in accordance with the WHO/ISUP criteria of 2004 [60]. All bladder cancer patients went through radical cystectomy or transurethral resection at the University Hospital Charité in Berlin between 2008 and 2009 and gave written informed consent for the use of representative tissue specimens for research purposes. The study was approved by the Ethic Committee of the University Hospital Charité (File: EA1/153/07).

### Isolation of RNA and Characterization of Quantity and Quality

The analyzed tumor tissues samples contained more than 80% tumor cells as previously described [59]. Approximately 20–30 mg of wet weight tissue was treated with 350 µl of lysis buffer and total RNA was isolated using the miRNeasy Mini Kit (Qiagen, Hilden, Germany) according to the manufacturer’s protocol. An additional DNase I digestion step on the RNA binding silica gel membrane was performed. The quantity and quality of isolated RNA was determined by a NanoDrop 1000 spectrophotometer (NanoDrop Technologies, Wilmington, DE, USA) and a Bioanalyzer 2100 (Agilent Technologies, Santa Clara, CA, USA). Only samples with RNA integrity number (RIN) values >5 were used. The RNA samples isolated from nonmalignant as well as from non-invasive and invasive tumor tissue samples showed comparable median 260/280 absorbance ratios (2.02, 2.03 and 2.03) and median RIN values (7.3, 6.7, and 7.2; Kruskal-Wallis test, P  = 0.486).

### Microarray-based RNA Analysis

miRNA expression analysis was performed by one-color hybridizations on human catalog 8-plex 15 K microRNA microarrays (AMADID 019118) from Agilent (Agilent Technologies, Santa Clara, CA, USA) which enclosed 723 human and 76 viral microRNAs from the Sanger miRBase (release 10.1). All reaction steps were carried out as previously described in detail [61]. After hybridization, microarrays were washed, scanned, and processed according to the supplier’s protocol. The raw data were normalized using Genespring GX11 Software (Agilent) with default parameters (threshold raw signal to 1.0, percent shift to 90th percentile as normalization algorithm and no baseline transformation). All microarray data has been deposited in the NCBI GEO database with accession number GSE36121.

mRNA expression analysis was performed by one-color hybridizations on whole human genome microarray 4×44 K v2 (026652) from Agilent comprising probes for human 34184 mRNA transcripts. After hybridization, microarrays were washed, scanned, and processed according to the supplier’s protocol. The raw data were normalized using Genespring GX11 Software (Agilent) with default parameters (percent shift to 75th percentile as normalization algorithm and a median baseline transformation of all samples). All microarray data has been deposited in the NCBI GEO database with accession number GSE40355.

### Classification of miRNA-mRNA interactions

#### miRNA-mRNA interaction data set

Validated human miRNA-mRNA interactions were obtained from Tarbase 5.0 and miRecords (version 11-2010) [34], [35], [62]. Human target mRNA predictions for miRNAs were extracted from TargetScan 5.2 and microRNA.org (version 8-2010) [22]–[25], [63]. The microRNA.org resource comprises predictions computed by the miRanda algorithm [30], [31]. In case of microRNA.org, the only predictions that were considered, were those annotated as ‘conserved miRNA’ and ‘good mirSVR score‘. For the analysis, the intersection between microRNA.org and TargetScan predictions was added to the set of validated interactions. miRNA families were extracted as defined in the TargetScan data set.

#### Algorithm for the classification of expression values

The goal of the algorithm is to partition the expression values corresponding to each probe into three sets: “high”, “medium” and “low”.

Let 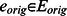 be the log-normalized expression value of a specific probe for a given sample which either refers to a miRNA or mRNA. 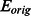 is the corresponding set of expression values of that probe over all samples. At first, the expression values are exponentiated, i.e. 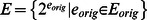. This way, we avoid some numeric issues. All values are larger than zero, because 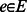 approaches zero as 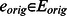 becomes more negative, i.e. when 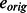 approaches 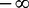, also, if 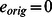 then 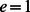. Clearly, there is a dependence on how the initial data was normalized.

We define the absolute fold change as 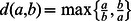 for two values 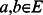. Please notice, that 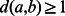.

There are two preliminary considerations. The first assumption is that two expression values are differentially expressed if their absolute fold change 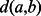 is higher than a certain threshold 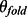. The second assumption is that values which absolute fold change is in a certain range are similarly expressed, i.e. their absolute fold change is lower than or equal to a threshold 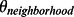.

Given 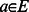 and a nonempty set *B* where 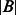 is the cardinality of set *B*, we define the absolute fold change between *a* and the mean of set *B* as 
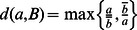
, where 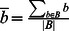. Again, 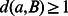 since *B* is nonempty, and 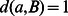 if and only if 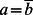.

We define that set *A* is the neighborhood of *a* if and only if 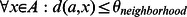 where 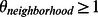.

We define *a* as representative of a set *A* if and only if *A* is the neighborhood of *a*. Please notice, that there can be more than one representative for a set *A*, i.e. for two values 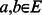 where *A* is neighborhood of *a* and *B* is neighborhood of *b,* if 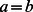, but also if 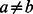 and 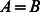.

We define a scoring function on two elements, *a* and *b* and their neighborhoods *A* and *B* as follows:
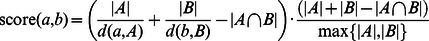



We add following constraint to determine the final score, where 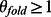 :
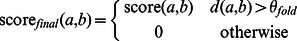



The rationale behind this scoring function is to find two sets of similarly expressed values which cover most of the data, thus also which overlap little as possible, i.e. the data coverage term 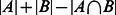. In addition, more equally sized sets are higher scored, i.e. the size distribution term 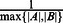. Otherwise one set could contain a single member and the other set all other members. Since, the data coverage should be more than linearly weighted compared to the size distribution of the sets, we introduced a quadratic relationship on the data coverage term. The last sort of terms, i.e. the set representative penalty terms 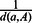, penalize set representatives that are far from their neighborhood. The set representative penalty terms should have less influence than the data coverage term, thus these terms are introduced into only one of the two data coverage terms.

To sum up the essential meaning of the scoring function, we identify two different neighborhoods, i.e. values of similar expression. These neighborhoods differ by at least a defined absolute fold change, but then the absolute fold change can be arbitrary large. The scoring function evaluates to what extend these neighborhoods are useful to represent the data, based on data coverage not absolute values.

Given the two resulting sets and their corresponding representatives which produce the highest final score, we denote the representative with the lower value as 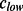 and the representative with the higher value as 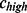. Based on 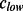 and 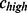, two boundaries 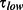 and 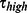 are calculated as follows:
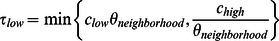


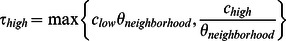



The rationale for this is as follows. The boundaries are defined by the upper limit of the lower set, and lower limit of the upper set; if the sets overlap, the boundaries are switched.

Finally, for each 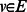 the classification of *v* is defined by:
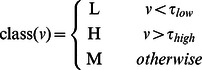



This classification will be referred to as state in the following.

For the actual classification of expression values, the fold threshold and neighborhood threshold is determined dynamically from a list of predefined paired values, i.e. a pair 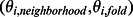 for the i-th element in the list. Separately for each miRNA or mRNA probe, the fold threshold and neighborhood threshold that yield the highest 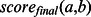 for that particular set of expression values are used. For this study, we defined 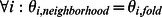.

#### Filtering and interaction states

Only those miRNA or mRNA probes are considered that exceed a certain score higher than a threshold 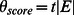 where *t* is an arbitrary real value and the cardinality 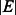 is the number of samples. Considering a single sample, mRNA probes which are mapped to the same EntrezGeneID are classified by the maximum occurring state. On a tie, the preferences for the classification are low (L), high (H) and then medium (M). Before interactions are classified, the mRNA and miRNA probes are filtered by the ratio of M classified samples, where 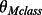 is the corresponding threshold. For a miRNA-mRNA interaction and for each sample the classification of an interaction is the combination of the two states of the miRNA and mRNA in that order, e.g. if a miRNA is classified as L for a specific sample and the target mRNA is classified as H, then the state of the interaction is LH. Hence, there are nine possible states for an interaction: *S*  =  {LH, HL, LM, HM, MH, ML, HH, LL, MM}.

We group these combinations by their biological meaning:

Down-regulated states *S_compHL_*  =  {HL, ML, HM}; up-regulated miRNA cause hypothetic down-regulation of mRNA.

Up-regulated states *S_compLH_*  =  {LH, MH, LM};. Down-regulated miRNA cause hypothetic up-regulation of mRNA.

Undefined states *S_undef_*  =  {HH, LL, MM} which do not follow the biological interpretation mentioned above.

Interactions with a frequency of undefined states 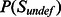 higher than a threshold 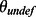 were excluded from the set of interactions. We will further refer to the set of interactions which satisfies the filtering criteria mentioned above as the set of regulated interactions.

Given two pre-defined groups *A* and *B*, it was defined that an interaction is differentially regulated for *A* and *B*, if the state with the maximum frequency of group *A* is an element of 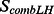 and the state with the maximum frequency of group *B* is an element of 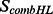 or reciprocal. For all data sets in this study, we set 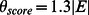, 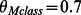 and 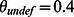.

#### Jaccard-Index

For each interaction, a Jaccard-index is calculated to evaluate the agreement between the predefined experimental groups 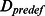 and the expected groups based on the assumption that an mRNA is down-regulated for one group and up-regulated for the other group by a specific miRNA.

Therefore, a partition 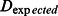 is computed where the samples are grouped into the three groups 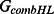, 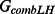 and 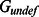. Where 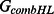 is the set of samples that have an interaction state of either HL, HM, or ML, 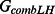 is the set of samples that have a interaction state of either LH, LM, or MH and 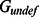 is the set of samples whose state is either HH, MM, or LL.

**Figure 1:**
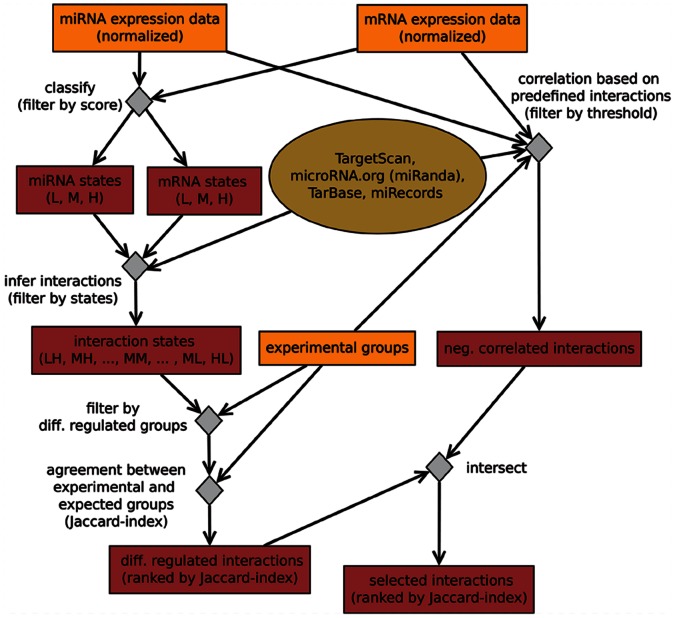
Flow chart for identifying differentially regulated interactions. Input data is depicted by orange rectangles. Output data is indicated by red rectangles. The ellipse refers to the set of inferred interactions. This set is independent of the input data, though it can be changed. Operations to manipulate data are depicted as diamonds.

The Jaccard-Index is then the similarity between the two partitions 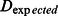 and 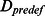 and assumes a value between 0 and 1 [64], [65]. Figure 1 summarizes the steps that were performed to identify differentially regulated interactions in this study.

#### Simple classification model

To evaluate the applicability of our ranking by Jaccard-indexes, based on the set of selected differentially regulated interactions a simple classification model is constructed which predicts the first group of an ensemble of samples, e.g. cancer samples from a collective of cancer and non-cancer samples.

Such a model contains a set of states 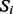 for each interaction *i* of the set of selected interactions *I*, where 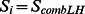 or 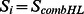. In addition, a set of undefined states 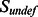 is defined. For each sample 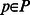, the sum of interactions classified as the first group is given by 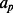 for all interactions *i* where the state of the sample 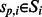. 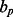 refers to the sum of interactions classified as the second group, i.e. all interactions *i* where the state of the sample 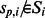 and 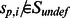. In other words for a sample, we increment 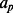 if the state of the sample indicates a regulation in the same direction as defined in the model for the specific interaction, we increment 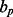 if the state of the sample corresponds to the opposite regulation and nothing is incremented if the state of the sample corresponds to an undefined interaction state. The classification of the sample is then defined by the maximum of 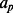 and 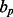.

A model is generated from the highest ranked interactions within a threshold value for the Jaccard-index of an interaction or by a defined number of randomized interactions within a range of Jaccard-indexes. The states 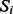 are defined according to the state with the highest frequency for the first group.

#### Bootstrapping analysis

The normalized expression values were randomly divided into training and test sets where each training set contains half of the samples of each group without replacement. If the number of samples was odd for a group, the trainings sets were assigned one sample more than the test sets for that group. Concerning the bladder cancer data set, for the collective of all samples, each training and each test set contains eight samples from either the group of invasive or non-invasive bladder cancer samples and four normal tissue samples. For the collective of bladder cancer samples, each training and each test set contains four invasive bladder cancer samples and four non-invasive bladder cancer samples. For the two collectives, 100 different data sets of training and test sets were generated by randomly dividing the samples under the constraints mentioned above. miRNA-mRNA interactions were computed and classified separately for each training and each test set. For each of the 100 data sets a model was computed based on the training set and applied to the corresponding test set. Mean specificities, sensitivities and false positives rates were computed over all 100 data sets.

Similarly to the bladder cancer data set, a colon cancer and prostate tumor data set that contain paired miRNA/mRNA micro array expression data was used to estimate specificities and sensitivities. Colon tissue samples and prostate tissue samples were extracted from the data set provided by Lu et al. [57] and treated as two separate data sets. In more detail, the colon tumor data set comprises four healthy samples and seven tumor samples. The prostate tumor data set contains six healthy and six tumor samples. For both the colon cancer and the prostate tumor data set separately, 50 randomized training sets and test sets were generated, then mean specificities and sensitivities were calculated in the same way as mentioned above.

In addition for the cancer tissue sample collective of the bladder cancer data set, the whole procedure was performed with an outlier removed and the same outlier re-assigned to the expected group according to the results of our examination.

### Prediction Analysis of Microarrays for R

To compare the results of our classifiers to another method, Prediction Analysis of Microarrays for R (pamr) [58], was performed using the same training and test sets as mentioned above. Pamr comprises a k nearest shrunken centroid classifier. A threshold value is used to define the extend of shrinkage for a model, i.e. a lower threshold value will generate a larger model and a higher threshold smaller model. Pamr was applied to each set of log-normalized miRNA and mRNA expression data separately. First we determined a range of thresholds separately for the miRNA and mRNA data of each data set by using ‘pamr.plotcv’ for some instances of training sets. Next, we used that range of thresholds to iterate over all randomized training sets corresponding to a miRNA or mRNA of a data set, computed the models and classified the corresponding test sets. ‘pamr.adaptthresh’ was used to rescale the model before classifying the corresponding test set. Except for the threshold default parameters were used for all functions of pamr.

Mean specificities and sensitivities were calculated in the same way as mentioned above.

### Correlation coefficients

For each of three experimental groups, i.e. invasive bladder cancer samples, non-invasive bladder cancer samples and normal tissue samples, Spearman correlation coefficients, ρ, were calculated between the miRNA and mRNA expression. The log-normalized expression values were used as input data. Pairs of miRNA-mRNAs were defined by the same set of interactions, as mentioned above. The expression values were treated separately for each of the three experimental groups. Spearman correlation coefficients were calculated for each pair of miRNA-mRNA interactions for each group.

### Processing of the bladder cancer data set

We applied our approach to two different collectives, a collective of all samples (8 non-invasive- and 8 invasive tumor samples as well as 8 control persons) and a collective of tumor samples with different tumor stages (8 non-invasive and 8 invasive samples) without healthy persons. For both collectives, only miRNAs and mRNAs expression values were processed showing in at least 20% of the used samples a “present call”, indicated by the microarray normalization software Genespring GX. Next, we applied our approach to identify differentially regulated interactions. In a further step, we selected only interactions that show a negative correlation, i.e. ρ≤−0.4, between normalized miRNA and mRNA expression values for at least one experimental group. For the collective of cancer tissue samples these groups are the invasive bladder cancer samples and non-invasive bladder cancer samples. For the collective of all samples the groups comprise both bladder cancer sample groups and the group of normal tissue samples, i.e. three different groups.

### Clustering

Based on the interaction states a principal component and clustering analysis was performed. For this purpose, the interaction states were substituted into real values as mentioned in Table 1. A distance matrix was computed using the city block distance as a metric. Afterwards, hierarchical clustering was performed using Ward’s method as a distance measure [66]. Principal components of the distance matrix were calculated where the distance matrix was treated as a set of *N N*-dimensional vectors [67].

**Table 1:** Substitution of interaction states into real values.

State	LH	MH	LM	LL	MM	HH	HM	ML	HL
Value	−1.0	−0.5	−0.5	0	0	0	0.5	0.5	1

### Functional annotation clustering

Genes which are involved in the differentially regulated interactions between miRNA and mRNA were analysed using the database for annotation, visualization and integrated discovery (DAVID) [15] with standard classification stringency parameters.

### Analysis of the bladder cancer data set using Magia2 and TaLasso

For comparative analysis, we applied four additional approaches to analyze the two collectives of bladder cancer samples, the collective of healthy and tumor samples and the collective of invasive and non-invasive tumor tissue samples. The TaLasso web server was used to identify miRNA-mRNA interactions with the TaLasso method and GenMiR++ algorithm [55]. The union between Tarbase, miRecoreds 2010 and the intersection of miRandaXL, PicTar 4-way and Targetscan (miRGen) was selected as set of putative miRNA-mRNA interactions.

In addition, Spearman correlations and a Meta analysis approach using the Magia2 web server were used to analyze the data sets [46]. For analysis with Magia2, the intersection between predictions from TargetScan and microRNA.org (miRanda) was defined as set of putative interactions. Concerning the analysis using Spearman correlation, only interactions are considered that exhibit a negative correlation, i.e. ρ <0.

For all approaches and both collectives, only miRNAs and mRNAs expression values were processed showing in at least 20% of the used samples a “present call”, indicated by the microarray normalization software Genespring GX. Log-normalized expression values were used for analysis, as mentioned above. When more than one probe was mapped to the same EntrezGeneID, for each sample, the median of the log-normalized expression values over the probes was calculated.

To determine the set of “best” interactions, interactions were ranked by highest score when TaLasso or GenMiR++ was used, by most negative correlation when Spearman correlations were calculated and by lowest q-value when the Meta analysis approach was used.

### Availability

An implementation of our algorithm as package of command-line tools is available at http://sourceforge.net/projects/caperna. It can be used under the terms of the GNU General Public License v3.0.

## Results

### Stratification of samples, PCA and hierarchical clustering

**Figure 2:**
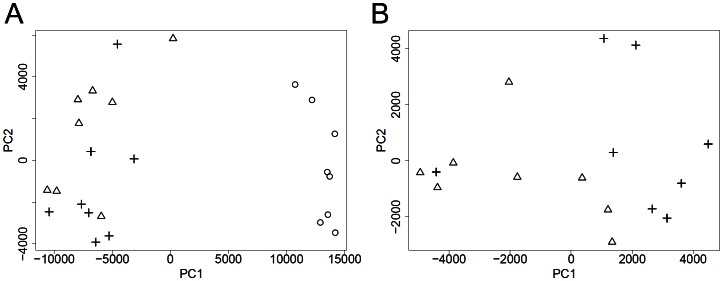
Spread of samples inside the bladder cancer data set. For the collective of all samples (A) and the collective of tumor samples (B). The first two principal components (PC) of the distance matrix based on interaction states are shown. Circles refer to samples of the control group, triangles are invasive tumor samples and crosses refer to samples of non-invasive tumors. The first two principal components explain 85.39% of the total variance (PC1 = 78.12% and PC2 = 7.27%) for (A) and 48.35% of the total variance (PC1 = 33.36% and PC2 = 14.99%) for (B).

After classification and filtering, our algorithm identified 11562 interactions for the whole collective of bladder tissue samples and 9075 interactions for the collective of bladder tumor samples that exhibit different states of regulation over the samples and assigned interaction states. At this stage, no a priori group knowledge is inferred. Distances between the samples were calculated using interaction states. Comparing the spread of samples in the first two principal components, one can clearly distinguish samples of the control group from tumor samples (Figure 2A). However, there is not a complete distinction between invasive and non-invasive tumor tissue samples, when the whole collective is analyzed.

Figure 2B shows the spread of structures for the non-invasive and invasive tumor tissue samples when they are analyzed separately from the control group. There is a separation between non-invasive and invasive tumor tissue samples, the group of non-invasive tumor samples tends to cluster together except for one outlier at the left side of the figure. For invasive tumor tissue samples, two clusters can be observed, one which is isolated from the group of non-invasive tumor tissue samples and a smaller cluster which is closer to the cluster of non-invasive tumor tissue samples.

**Figure 3:**
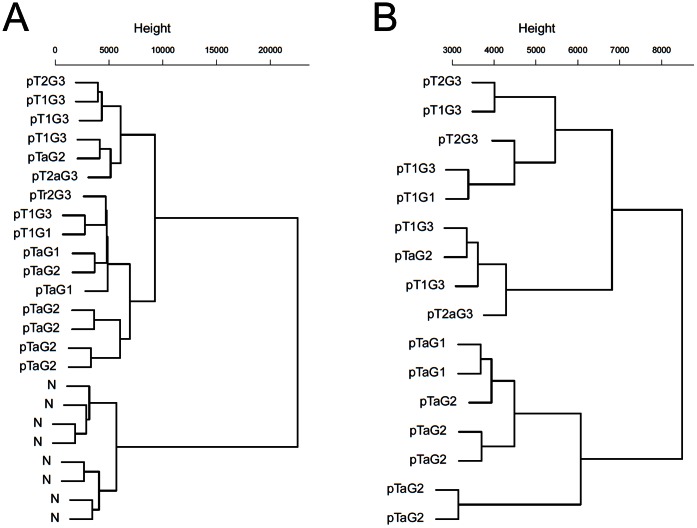
Hierarchical clustering of the bladder cancer data set based on interaction states. For the whole collective of samples (A) and the collective of tumor samples (B) using interaction states computed by algorithm. “N” refers to samples of the control group. Tumor samples were labeled by their pathological staging (non-invasive: pTa or invasive: pT1 and higher).

A cluster analysis reveals similar results. Samples of the control group form an isolated cluster that is far from the groups of tumor samples (Figure 3). Concerning the collective of non-invasive and invasive tumor tissue samples, the non-invasive tumor samples form a single cluster, except for the outlier mentioned above (Figure 2). A cluster of three invasive tumor samples is closer to the cluster of non-invasive tumor samples while the larger cluster of invasive tumor samples shows a bigger distance to the non-invasive tumor samples. The outlier is part of the larger cluster of invasive tumor samples.

### Bootstrapping analysis

As identifying altered miRNA-mRNA interactions between two groups of tissues is a form of feature selection we argue that a valid ranking of putative miRNA-mRNA interactions should be reflected by the selected features. The quality of selected features can be measured by the performance of classifiers constructed from these features. We have generated randomized non-overlapping trainings and test sets for four different collectives, I. healthy bladder cancer tissue and bladder tumor tissue, II. non-invasive bladder tumor tissue and invasive bladder cancer tissue, III. healthy colon tissue and colon tumor samples and IV. healthy prostate tissue samples and prostate cancer samples. Next, we created simple models based on the interactions with the highest Jaccard-indexes using our method, CAPE RNA, and trained models on the same sets, but separately for miRNAs and mRNAs, using Prediction Analysis of Microarrays for R (pamr) [58]. For this part of the analysis, when our method was used, we filtered bladder cancer data by present call, but did not filter interactions by negative correlation for any data set. Mean values for specifities and sensitivies were calculated for both types of classifiers.

**Figure 4:**
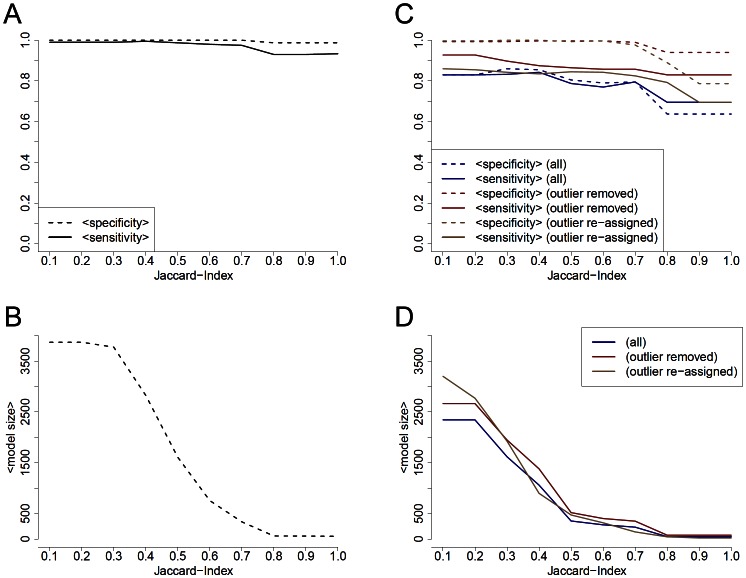
Mean specificities and sensitivities for the bladder cancer data set using our algorithm CAPE RNA. Models for the whole collective (A) and only for tumor samples (C) were generated from training sets by selecting all interactions with a Jaccard-index equal to or higher than a threshold. A and C illustrate the mean specificities and mean sensitivities for the models to classify an unknown test set. B (generated from the whole collective) and D (only tumor samples) shows the average number of interactions included in a model.

**Figure 5:**
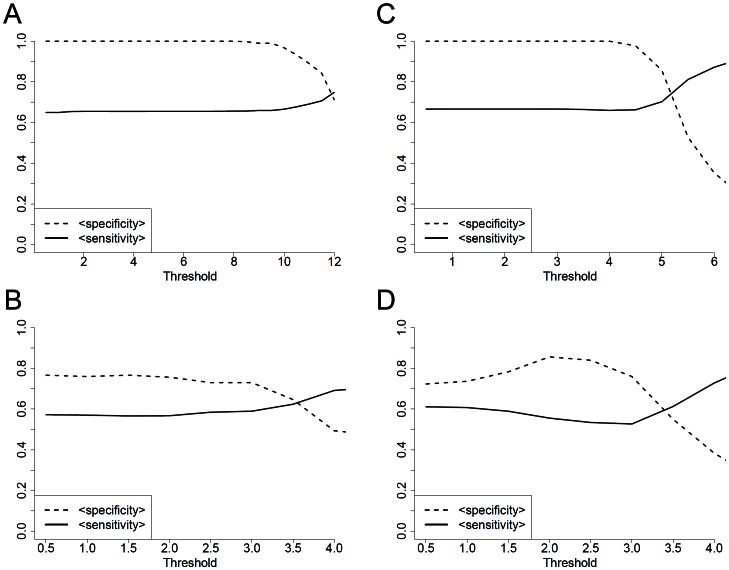
Mean specificities and sensitivities for the bladder cancer data set using pamr. Prediction Analysis of Microarrays for R was used to train models for the collective of all samples based on miRNA expression (A) and mRNA expression (C), as well as for the collective of tumor samples based on miRNA (B) and mRNA (D). Models were generated with different thresholds. A-D) illustrate the mean specificities and mean sensitivities to classify unknown test sets.

For collective I, our method exhibits mean specificities between 0.99–1 and mean sensitivities between 0.93–0.99 depending on the lowest allowed Jaccard-index, thus the size of the model, Figure 4A, 4B. In comparison, models generated by pamr achieve specificities up to 0.9–1, but in that range of thresholds sensitivities between 0.65–0.70, Figure 5A, 5C. Concerning collective II, we identified an outlier performing PCA and using our approach. Figure 4C shows the expected specificities and sensitivities including the outlier, without the outlier and when the outlier is reassigned from non-invasive cancer to invasive cancer. There is an increase in the mean specificities up to 0.2 when the outlier is removed or reassigned, also there is a relevant increase in the sensitivities. For this reason we removed the outlier for the pamr analysis. Using pamr, mean specificities lie between 0.72–0.86 and sensitivities between 0.61–0.51 when using mRNA expression data. Mean specificities increase up to 0.77 when miRNA expression data is used, however there is a decrease in mean sensitivities, Figure 5C, 5D. In contrast, our approach exhibits mean specificities between 0.94–0.99 and mean sensitivities between 0.83–0.93.

For the colon and prostate collectives (III, IV), our method shows mean specificities between 0.89–1 and mean sensitivities between 0.87–0.97, Figure S3A,B. The pamr classifier exhibits mean specificities up to 0.72–0.96 and mean sensitivities below 0.6 in that threshold region for the miRNA expression data of collective IV, Figure S3D. Concerning collective III and IV, there is no threshold where both mean specificities and mean sensitivities are above 0.7 when pamr is used with either mRNA or miRNA expression data, Figure S3C-F.

### Differentially regulated interactions in bladder cancer

**Figure 6:**
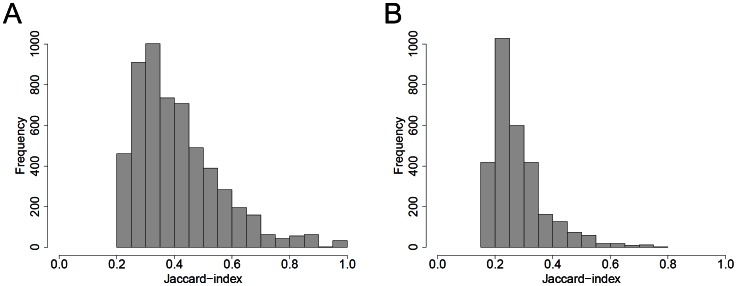
Distribution of Jaccard-indexes for the bladder cancer data set. For the whole collective (normal and tumor samples) (A) and the group of tumor samples (invasive and non-invasive tumors) (B). Jaccard-indexes were calculated using our approach. Only Jaccard-indexes corresponding to interactions which show a negative correlation, i.e. ρ≤−0.4, for at least one experimerntal group between normalized miRNA and mRNA expression values are depicted.

We have examined the miRNA-mRNA interactions inside the bladder cancer data set more elaborately. The outlier which has been identified by PCA was removed for analysis of the non-invasive and invasive bladder tumor collective. After inferring a priori knowledge of the groups and selecting interactions by interaction states that indicate a differential regulation between the two groups, the set of selected differentially regulated interactions contains 9180 interactions for the collective of all samples and 5963 differentially regulated miRNA-mRNA interactions for the collective of tumor samples. Next, the interactions were selected that exhibit a negative correlation between miRNA and mRNA expression. There were 5583 interactions for the collective of all samples and 2938 interactions for the collective of cancer samples that satisfy these criteria. Compared to these numbers, there are relatively few interactions which are suitable to partition the samples into their pre-defined groups when used as single interaction, i.e. interactions that exhibit a high Jaccard-index (Figure 6).

For the collective of all samples, there are 29 interactions which yield a perfect classification of the collective as single interaction, Table 2. miR-429 and miR-200c are involved in all of these, except for one interaction which includes miR-183. A common feature of all the interactions is that the miRNAs are upregulated in cancer tissue. These are merely likely examples of potential interactions. The list of differential regulations can be extended by several interactions with a less perfect Jaccard-index.

**Table 2:** Differentially regulated interactions between healthy and tumor samples for the bladder cancer data set.

miRNA	miRNA-expression	Gene Symbol	geneID	Gene-expression
miR-200c	Up	ABCC9	10060	Down
miR-429	Up	ABCC9	10060	Down
miR-429	Up	AKAP2	11217	Down
miR-200c	Up	DDIT4L	115265	Down
miR-429	Up	DDIT4L	115265	Down
miR-200c	Up	DMD	1756	Down
miR-429	Up	DMD	1756	Down
miR-429	Up	ZCCHC24	219654	Down
miR-429	Up	FOXF1	2294	Down
miR-429	Up	FYN	2534	Down
miR-200c	Up	MYLK	4638	Down
miR-429	Up	MYLK	4638	Down
miR-429	Up	NCAM1	4684	Down
miR-200c	Up	ROR2	4920	Down
miR-429	Up	ROR2	4920	Down
miR-200c	Up	RAB23	51715	Down
miR-200c	Up	PLCL1	5334	Down
miR-429	Up	PLCL1	5334	Down
miR-183	Up	SOBP	55084	Down
miR-429	Up	ARHGAP20	57569	Down
miR-200c	Up	KIAA1462	57608	Down
miR-429	Up	KIAA1462	57608	Down
miR-200c	Up	REEP1	65055	Down
miR-429	Up	REEP1	65055	Down
miR-200c	Up	ZEB1	6935	Down
miR-429	Up	ZEB1	6935	Down
miR-429	Up	C7orf58	79974	Down
miR-429	Up	RECK	8434	Down
miR-429	Up	DIXDC1	85458	Down

Only, interactions were selected that exhibit a negative correlation, i.e. ρ≤−0.4, between the normalized miRNA and mRNA expression values for at least one experimental group. Interactions with a Jaccard-index equal to 1.0 are shown. The regulation of a gene inside the group of the bladder cancer samples compared to the normal tissue samples is indicated.

For the differences in regulation between the non-invasive and invasive tumors, there are no interactions with a perfect Jaccard-index of 1. However, there are twelve interactions with a Jaccard-index higher than 0.70, see Table 3. These interactions include miR-7, miR-24, miR-26b, miR-29b, miR-29c and miR-30b. A list of all selected differentially regulated interactions and their Jaccard-indexes for both collectives is available as supplementary data, Table S1 and Table S2, as well as a list of the corresponding Spearman correlation coefficients of interactions used for filtering, Table S3 and Table S4.

**Table 3:** Differentially regulated interactions for non-invasive and invasive bladder tumor tissue samples.

miRNA	miRNA-expression	Gene Symbol	geneID	Gene-expression	Jaccard-index
miR-26b	Down	DEPDC1	55635	Up	0.76
miR-29c	Down	C20orf11	54994	Up	0.75
miR-29b	Down	DOLPP1	57171	Up	0.75
miR-26b	Down	GNPNAT1	64841	Up	0.75
miR-29b	Down	PXDN	7837	Up	0.75
miR-29c	Down	PXDN	7837	Up	0.75
miR-29b	Down	MEX3B	84206	Up	0.75
miR-29c	Down	MEX3B	84206	Up	0.75
miR-26b	Down	NUP153	9972	Up	0.75
miR-7	Up	NRSN1	140767	Down	0.72
miR-30b	Down	RTKN2	219790	Up	0.72
miR-24	Down	CDK1	983	Up	0.72

Only, interactions were selected that exhibit a negative correlation, i.e. ρ≤−0.4, between the normalized miRNA and mRNA expression values for at least one experimental group. An outlier has been removed before this analysis. Interactions with a Jaccard-index >0.70 are shown (rounded to the second decimal). The regulation of a gene inside the group of the invasive bladder cancer samples compared to the group of non-invasive cancer samples is indicated.

### Pathway based analysis of differentially regulated interactions

By pathway enrichment analyses we could show that corresponding genes of the detected miRNA-mRNA interactions were strongly associated with different KEGG cancer pathways including bladder cancer. The detailed list can be found in Table S5.

In this section, we would like to analyze two selected pathways in more detail. First, the growth factor receptor signaling pathway in bladder cancer (FGFR3), as defined in the review by Fendler et. al, is examined [12]. Second, important interactions in the bladder cancer pathway, as defined by KEGG, are investigated [68].

**Figure 7:**
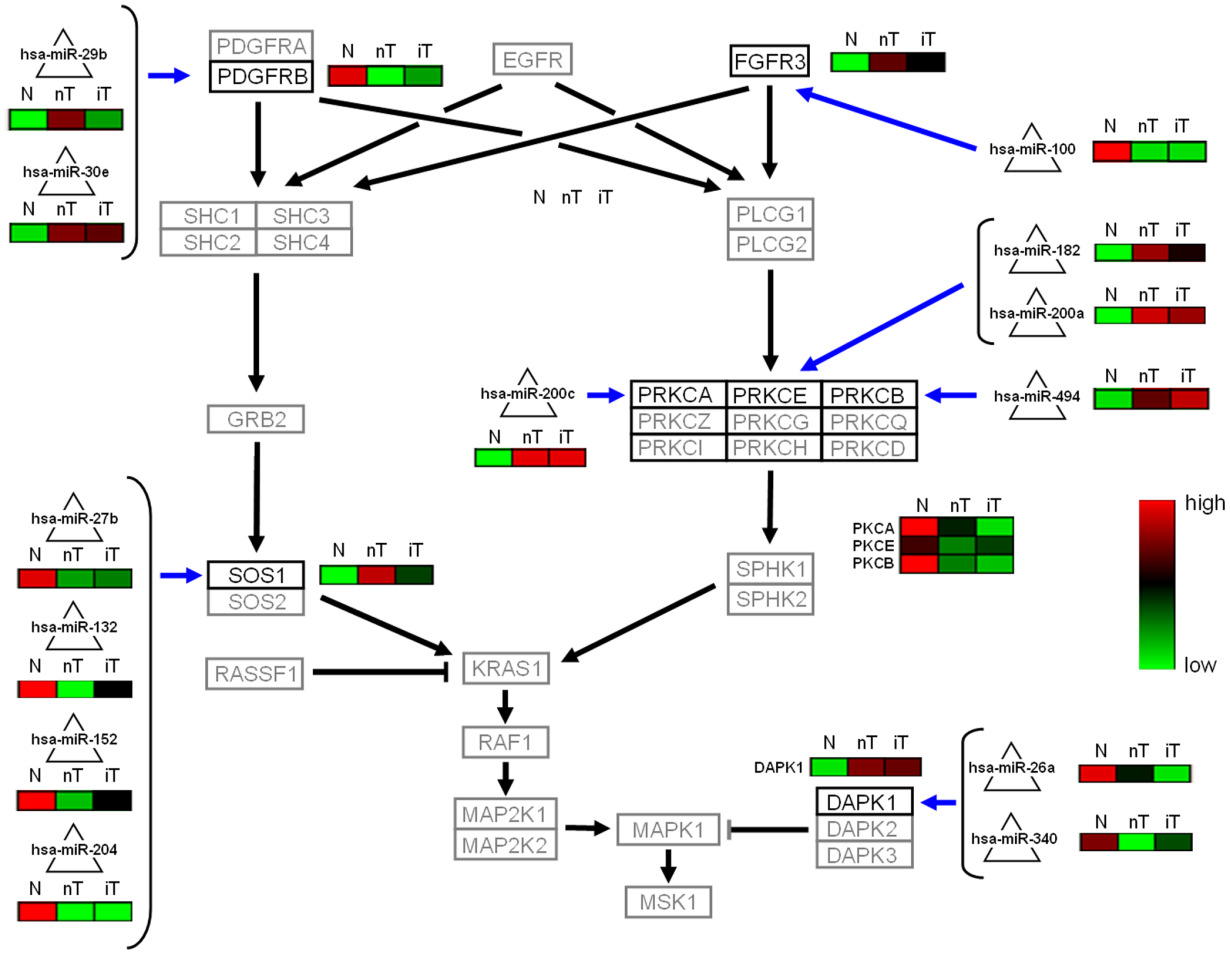
FGF3R pathway: detected miRNA-mRNA interactions in normal urothelium (N), non-invasive- (nT) and invasive (iT) bladder tumor tissue samples. Interactions between miRNAs (triangles) and mRNAs (boxes), which have a Jaccard-index ≥0.4 that exhibit a negative correlation, i.e. ρ≤−0.4, between the normalized miRNA and mRNA expression values for at least one experimental group, are shown (blue edges). The black edges indicated the general signal cascade. Mean expression status of the analysed miRNA mRNAs interactions were indicated (red indicates up regulation and green down regulation).

For both pathways, we selected only interactions with a Jaccard-index equal to or higher than 0.4 that were selected by negative correlation between miRNA and mRNA expression. Based on these criteria, we have identified 13 differentially regulated interactions in the FGFR3 pathway (Figure 7 and Table S6). In bladder cancer, FGFR3 mRNA expression is hypothetically up-regulated by miR-100 down-regulation. The alpha subunit of the protein kinase C (PRKCA) is hypothetically down-regulated by miR-200c. The miRNAs miR-200a and miR-182 down-regulate the epsilon subunit of the protein kinase C (PRKCE) while PRKCB is hypothetically down-regulated by miR-494. DAPK1 is hypothetically up-regulated due to lower expression levels of miR-26a and miR-340. In addition, our results suggest an up-regulation of SOS1 by miR-27b, miR-132, miR-152 and miR-204.

**Figure 8:**
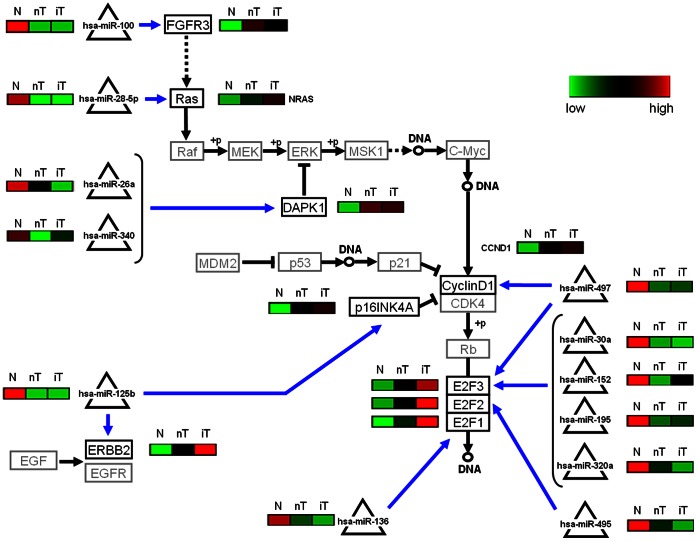
KEGG bladder cancer pathway: calculated miRNA-mRNA interactions differences between normal urothelium (N) and non-invasive- (nT) and invasive (iT) bladder tumor tissue samples. Interactions between miRNAs (triangles) and mRNAs (boxes), which have a Jaccard-index ≥0.4 that exhibit a negative correlation, i.e. ρ≤−0.4, between the normalized miRNA and mRNA expression values for at least one experimental group, are shown (blue edges). The black edges indicated the general signal cascade adopted from KEGG bladder cancer pathway. Mean expression status of the analysed miRNA-mRNA interactions are indicated (red indicates up regulation and green down regulation).

For the KEGG bladder cancer pathway there are sixteen interactions that satisfy the conditions (Figure 8 and Table S7). In addition to the up-regulation of DAPK1 and FGFR3 in the cancer tissue, our analysis suggests following interactions. Cyclin D1 (CCND1) is hypothetically up-regulated due to lower expression levels of miR-497. Also several members of the E2F transcription factor family are up-regulated due to lower expression levels of miR-30a, miR-152, miR-195, miR-320a, miR497 (all target to E2F3), miR495 (target to E2F2) and miR-136 (target to E2F1).

Furthermore, the oncogenes NRAS and ERBB2 are up-regulated in bladder cancer, hypothetically, due to lower of expression of miR-28-5p and miR-125b, respectively.

## Discussion

### On the validation of computational methods

In general to assess the validity of a prediction method, one could evaluate its performance on a data set where the correct result is known for every instance, then, for example, specificities and sensitivities can be calculated. For miRNA-mRNA prediction methods this proves very problematic because there is not such a data set. In addition, even experimental results may have a bias due to the researchers’ interpretation and may have a limited reproducibility. Furthermore, it is almost impossible to create such a data set in the first place, because it would, for instance, involve knockout experiments for every predicted interaction and every non-predicted interaction. Nonetheless, one can look at the problem from a different point of view. We stated earlier in this manuscript that predicting miRNA-mRNA interactions is a form of feature selection and one can at least evaluate the validity of selected features in the sense that they can generate strong classifiers. This does not imply that these interactions do actually occur in biological systems, but one may at least suggest that they are more likely to have a biological meaning. An important aspect is that one evaluates the performance to classify unknown instances, i.e. training sets and test sets must be separated, otherwise a classifier could be over-fitted and learn the data by heart. We argue that the method for the evaluation of the performance of a predictor for miRNA-mRNA interactions as presented by this study is more general and may be more reasonable than calculating statistics over a data set itself or comparing a limited number of literature results.

### Validation of our method and assumptions

For our approach, CAPE RNA, following observations may indicate its validity. The validity of interaction states is reflected in the results of the bootstrapping analysis since interaction states are the foundation of further steps in our approach. In addition, the results of the PCA and cluster analysis suggest they perform more reasonable to discriminate between the different groups of samples than the log-normalized expression data, Figure 2, Figure 3, Figure S1 and Figure S2. In particular, evidence for this qualitative interpretation is reflected in the bootstrapping analysis when the outlier is removed or reassigned, Figure 4C.

The results of the bootstrapping analysis show that a simple model based on our approach and assumptions outperforms a standard method for gene expression as a classifier. Furthermore, our method is applicable to very small sample sizes, i.e. for the prostate cancer collective a training set contains three samples for each group and for the colon cancer collective four tumor samples and two healthy samples.

It was our assumption that the Jaccard-index can be used as a ranking for interactions. To examine whether there is a relationship between the performance of classifiers and Jaccard-indexes independently of the size of the model, we generated models by randomly selecting a defined number of interactions within a range of Jaccard-indexes using the bladder cancer training and test sets (collective I and collective II) and evaluated their performance. Figure S4 presents evidence for our assumption.

Also, instead of randomly determining relationships between miRNAs and mRNA we used a set of putative miRNA-mRNA interactions which has a scientific basis. Furthermore, the genes involved in predicted interactions are enriched in cancer pathways.

### General comments on our approach

In this study, we present an approach that introduces a high level of abstraction by classifying expression data in three different states. Consequently, there is a loss of information. However, our method has several advantages. As gene expression data is very noisy data in general, the classification of data into few discrete states makes it much more feasible to analyze. The classification closely follows a biological assumption that there exist two groups, one group which exhibits a low expression level and another one which exhibits a high expression level, thus reducing the information content to a reasonable level of abstraction. It provides a simple way to classify miRNA-mRNA interactions into a small number of states that can be grouped by their biological meaning in down-regulated, up-regulated and undefined interactions as direct interpretation of siRNA mechanism. Using interaction states, individual differences between samples can be compared for a selected set of genes. There is no other approach known to us which provides such intuitive classifications for individual samples.

The scoring function for the classification depends on data coverage. Hence, outliers have almost no influence on the classification of the expression data of the other samples. Instead of using predefined groups, our algorithm identifies two groups, i.e. neighborhoods of similarly expressed values, by the data itself and gives a score which describes how much the two groups represent the expression values for a given probe. For other purposes, this scoring function could be generalized to multi-class problems, e.g.
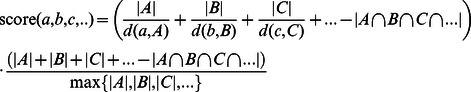



However, we are uncertain whether it would perform as well as for a two class problem. In addition, the combinatorial complexity increases with the number of classes. Especially, for paired data the number of interaction states increases quadratically and one would compromise the simplistic interpretation of those states. For differential regulation between two groups of samples, we are only interested in a two class problem.

Compared to most approaches based on statistical tests, e.g. a t-test, our method does not compare mean or median values of groups which may underestimate the regulation of single samples and also is error-prone to outliers. Our approach evaluates miRNA-mRNA expression data on the level of interactions. While the evaluation of miRNA and mRNA expression data as separate sets needs not be meaningful, one can identify reasonable differences in regulation by examining the combined set of miRNA-mRNA interaction states.

The examination of differential expression levels alone may be insufficient to identify direct interactions between miRNAs and mRNAs. On the other hand, biological systems are very complex systems. Several miRNAs may target the same mRNA and other factors such as transcription factors may influence the expression of a specific mRNA. Therefore, one needs not necessarily find a strong negative correlation between the expression levels of a miRNA and the target mRNA. However, the identification of even low or moderate negative correlation between miRNA and mRNA expression increases the certainty of a predicted interaction.

The augmentation of our approach by testing for negative correlation, is a reasonable choice not to over- or underestimate differential expression levels and direct correlation between miRNA and mRNA expression.

### Comparison to other approaches

We have compared our approach, CAPE RNA, to four related methods, TaLasso, GenMiR++, Spearman correlation and a Meta analysis approach. The TaLasso web server offers an implementation of both TaLasso and GenMiR++ [55]. TaLasso is a method based on LASSO regression whereas GenMiR++ exploits a Bayesian learning approach [51]. Spearman correlation coefficients and the Meta analysis approach were computed using the Magia2 web server [46]. For TaLasso, GenMiR++ and Spearman correlation paired data is analyzed, but no information about different groups of samples, e.g. healthy tissue and disease samples, can be specified. The Meta analysis approach as implemented in Magia2, does not consider paired sample labels, but needs information about the different groups which the samples belong to. The Meta approach calculates significant differential expression between the given groups of samples separately for each miRNA and mRNA using LIMMA [69] and identifies oppositely expressed miRNA-mRNA pairs by a chi-square test. Other tools for integrative analysis of miRNA/mRNA expression data include miRConnX [49] which computes different correlation coefficients and MMIA [47] which uses a similar method compared to the Meta approach, mentioned above, based on a t-test. In addition, Magia2 offers a mutual information based approach, but this approach is limited to a sample size equal to or larger than 20, thus, it could only have been applied to one of the two analyzed collectives.

To compare the four different methods mentioned above and our approach, we formulated a simple problem: given a data set of paired miRNA/mRNA expression values and two known groups of samples give us the 500 most promising candidates for interesting miRNA-mRNA interactions. For this purpose, we analyzed the collective of healthy tissue samples and bladder tumor samples (collective I) and the collective of invasive and non-invasive bladder cancer samples (collective II).

Although similar sets of potential interactions were used, the results of this comparison are clearly biased since the set of putative interactions differ for different approaches. The Meta analysis and correlation based analysis were performed with the same set of interactions. For TaLasso and GenMiR++ the same set of interactions was used, but for our approach another set of interactions was used. This is a general problem when different tools for the identification of miRNA-mRNA interactions are compared.

**Figure 9:**
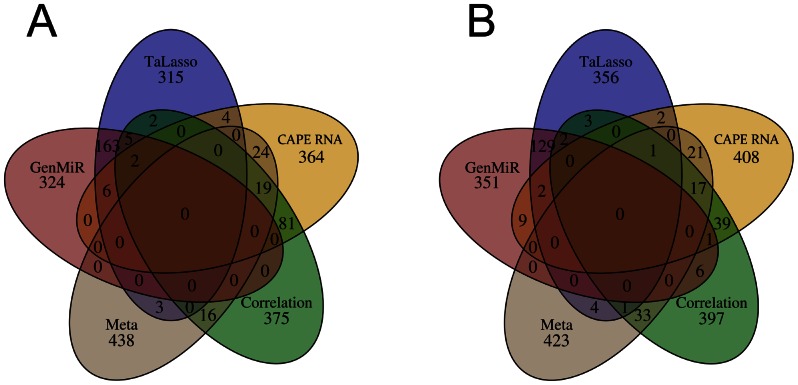
Venn diagram of predicted miRNA-mRNA interactions in bladder cancer derived from five different methods of integrative analysis (TaLasso, GenMiR++, Spearman correlation, a Meta analysis approach and our new algorithm CAPE RNA). Two different collectives were analysed: (A) the entire bladder cancer dataset of normal (n = 8) and tumor samples (n = 16) and (B) only the collective of invasive (n = 8) and non-invasive (n = 8) bladder cancer tumor samples. The Venn diagram illustrates the intersection between the top 500 predicted miRNA-mRNA interactions by each method.

Our results suggest that there is little overlap between predictions for the each best 500 interactions when different approaches are applied (Figure 9, Figure S5 shows the intersections between all predicted interactions). TaLasso and GenMiR++ share 176 (35.2%) and 133 (26.6%) interactions in the top 500 for collective I and collective II, respectively. Surprisingly, there are only seven (1.4%) to nine (1.8%) interactions overlap between interactions identified by correlation and TaLasso or GenMiR++. Our approach has most interactions in common with those identified by correlation, 102 (20.4%) and 58 (11.6%) interactions, and by the Meta approach, 43 (8.6%) and 39 (7.8%) interactions, but shares only five (1.0%) to twelve (2.4%) interactions with TaLasso and GenMiR++ in the top 500 (the list of the shared interactions is found in Table S8).

As for GenMiR++, TaLasso and Spearman correlation the data set is analyzed as a whole, they do not take differential expression between two groups into account. Therefore, we do not expect them to exhibit the same results as a method that considers differential expression does. Compared to our method, the Meta approach considers differential expression between groups, but does not analyze paired miRNA and mRNA expression per sample. Since we apply correlation as a filtering criterion and our method may show some similarities to correlation in general, we expected an overlap between analysis based on correlation and our approach. However, there are several differences between both methods. We calculate correlation for each experimental group separately and not over the data set as a whole which may be more reasonable when one considers a complex pattern of de-regulation and perhaps also loss or gain of function. Obviously, the main difference is our procedure in general. We consider differential expression and evaluate to what extent the partition of samples into expected classes resembles the defined groups. Concerning the relationship between all samples, our approach is less strict than correlation over a data set as a whole, but it emphasizes on the distribution of each sample based on paired miRNA-mRNA expression.

In conclusion, most approaches address similar problems, but not a two class problem for differential miRNA induced regulation, e.g. between healthy and disease tissue samples. Even when methods are applied that address the same problem, there is much less overlap between the top ranked interactions than one might have expected. Although there is a bias introduced by the set of potential interactions, this observation remains valid, e.g. when TaLasso and GenMiR++ are compared which share the same set of interactions. Correlation analysis, TaLasso and GenMiR are interesting methods to identify interactions in general or to analyze time series data while methods like the Meta approach most likely do not put enough emphasis on the regulation of individual samples. With CAPE RNA, we present a method that combines both differential expression and paired miRNA-mRNA regulation per sample. None of the methods or tools yields an individual classification of each sample similar to the interaction states based on our approach. Our method to evaluate the distribution of samples based on the agreement between expected and predefined (experimental) groups is unique among those approaches, as far as we know.

### Important differentially regulated interactions in bladder cancer

Among the highest ranked differentially regulated interactions we found several genes which are known to be involved in bladder tumor progression. For instance, the MMP repressor RECK (in our analysis hypothetically down-regulated in cancer by miR-429), which decrease was found associated with more invasive forms of bladder cancer [4].

One family of miRNAs appears to be most important for the differential regulation in bladder cancer, the family of miR-200b/c and miR-429. Other highly ranked interactions (Jaccard-index >0.8, 149 interactions in total), include miR-1,miR-143, miR-145, miR-183, miR-19a, miR-19b, miR-200a, miR-200b, miR-200c, miR-204,, miR-222, miR-23b, miR-33a, miR-425, miR-429. There are 88 miRNAs involved in 2480 interactions with a Jaccard-index equal to or higher than 0.4, and 25 miRNAs involved in 252 interactions with a Jaccard-index of at least 0.7. These numbers show us that there is a large network of differentially regulated potential interactions of which several different interactions may play in important role for tumorgenesis.

To distinguish invasive from non-invasive bladder cancer, 319 interactions with a Jaccard-index equal to or higher than 0.4 remain involving 34 miRNAs, but there are just twelve interactions with a Jaccard-index equal to or higher than 0.7 that comprise six miRNAs, namely miR-24, miR-26b, miR-29b, miR-29c, miR-30b and miR-7. These six miRNA are the most promising candidates to investigate tumor progression to invasive bladder cancer based on our examination.

### miRNA-mRNA interactions in selected pathway

miRNA-mRNA interactions in two selected pathways, were analyzed in more detail. First the FGF3R pathway as defined in the review by Fendler et. [12] and second the bladder cancer pathway provided from KEGG [68]. In the FGF3R pathway, DAPK1 mRNA coding a serine/threonine kinase whose expression is required for interferon-gamma-induced apoptosis. Although, DAPK1 promoter hypermethylation was previously described as an early alteration leading to bladder cancer [70], but no correlation between promoter methylation and mRNA expression was found [71]. In our dataset, DAPK1 mRNA was found up regulated in the tumor tissues, which is also supported from previous expression profiling in bladder cancer [72] (GEO-data: GDS1479). Interestingly, the DAPK1 expression is strongly regulated in our model by miR-26a and miR-340, respectively (Figure 7). We suspect, that the postulated hypermethylation-mediated reduction of DAPK1 mRNA expression in bladder cancer is more than compensated by the mRNA-miRNA interfering effect caused by the reduction of DAPK1 interfering miRNAs. The role of DAPK1 as an inactivated tumor suppressor gene in bladder cancer should be reconsidered. Another player in the FGF3R pathway is protein kinase C, represented by several different isoforms (Figure 7). In accordance to previous publications [73], [74] we found PKRCA and PKRCB mRNA down regulated in bladder cancer. We suppose that PKRCA mRNA reduction was caused by miR200c and PKRCB mRNA reduction by miR-494. Further, we found mRNA-miRNA interactions between PKRCE and two different miRNAs (miR-182, miR-200a).

In KEGG bladder cancer pathway, the oncogenes NRAS and ERBB2 were found up-regulated, hypothetically, due to lower of expression of miR-28-5p and miR-125b, respectively. The up-regulation of cyclin D1, which is involved in cell cylcle progression, is probably promoted by down regulation of miR-497. The Cyclin D/CDK4 complex phosphorylates pRB allowing it to disassociate from the transcription factor E2F. As previously reported [75], we also detected E2F3 as over expressed in bladder cancer. In our model miR-30a, miR-152, miR-195, miR320a and miR-497 promote the expression of E2F3, respectively (Figure 8).

### Limitations

In this study, the identification of interactions is limited by the used set of validated and predicted miRNA-mRNA interactions and by our approach in the sense that we only consider probes which exhibit two distinct expression levels based on our definition. Also, the certainty of identified differentially regulated interactions depends on the number of samples and the distribution of samples over the two groups. Based on the definition of our scoring function, this approach is unlikely to perform well if the number of samples differs too much between the two groups. Although we used negative correlation between miRNA and mRNA expression as filtering criterion, one cannot rule out the influence of other factors on gene expression such as transcription factors.

While the identified interactions present many interesting candidates for biomarkers, the underlying mechanisms can only be understood partially by the examination of a limited of subset of genes. The analysis of the FGF3R and bladder cancer pathway indicates a few differentially regulated switches. A more elaborate understanding is adjourned to future examinations.

## Supporting Information

Figure S1 **Spread of samples for the bladder cancer data set using normalized expression values.** The first two principal components (PC) of the normalized data are shown for the collective of all samples (A and C) and the collective of tumor samples (B and D) using normalized miRNA (A and B) or mRNA (C and D) expression data, respectively. Circles refer to samples of the control group, triangles are samples revealed invasive tumors and crosses refer to samples with non-invasive tumors.
(TIF)

Figure S2 **Hierarchical clustering of the bladder cancer data set using normalized expression values.** Hierarchical clustering of the normalized expression data was performed using Ward’s method for the collective of all samples (A and C) and the collective of tumor samples (B and D) using normalized miRNA (A and B) or mRNA (C and D) expression data, respectively.
(TIF)

Figure S3 **Mean specificities and sensitivities for the colon and prostate cancer data.** Models for the colon collective (A) and prostate collective (B) based on our approach, CAPE RNA, were generated from training sets by selecting all interactions with a Jaccard-index equal to or higher than a threshold. Prediction Analysis of Microarrays for R was used to train models for the colon tissue samples based on miRNA (C) and mRNA expression (D), as well as for the prostate collective based on miRNA (E) and mRNA (F) expression. Models were generated with different thresholds. A-F) illustrate the mean specificities and mean sensitivities to classify unknown test sets.
(TIF)

Figure S4 **Mean specificities and sensitivities for the bladder cancer data set by randomized models.** Models were generated by randomly picking a defined of interactions within a specific range of Jaccard-indexes using our approach, CAPE RNA. The performance of different models was compared to classify unknown test sets: a) specificities and b) sensitivities to discriminate tumor samples from healthy tissue samples, c) specificities and d) sensitivities to discriminate invasive tumor samples from non-invasive tumor samples.
(TIF)

Figure S5 **Venn diagram of predicted miRNA-mRNA interactions in bladder cancer derived from five different methods for integrative analysis (TaLasso, GenMiR++, Spearman correlation, a Meta analysis approach and our new algorithm CAPE RNA).** Two different collectives were analysed: (A) the entire bladder cancer dataset of normal (n = 8) and tumor samples (n = 16) and (B) only the collective of invasive (n = 8) and non-invasive (n = 8) bladder cancer tumor samples. The number of all predicted miRNA-mRNA interactions by each method were visualized in the Venn diagram.
(TIF)

Table S1 **List of all selected differentially regulated interactions and their Jaccard-indexes for all samples for the bladder cancer data set.** Only, interactions were selected that exhibit a negative correlation, i.e. ρ≤−0.4, between the normalized miRNA and mRNA expression values for at least one experimental group.
(CSV)

Table S2 **List of all selected differentially regulated interactions and their Jaccard-indexes for non-invasive and invasive bladder tumor samples.** Only, interactions were selected that exhibit a negative correlation, i.e. ρ≤−0.4, between the normalized miRNA and mRNA expression values for at least one experimental group.
(CSV)

Table S3 **Spearman correlation coefficients of all interactions for all samples of the bladder cancer data set.** Only, interactions were selected that exhibit a negative correlation, i.e. ρ≤−0.4, between the normalized miRNA and mRNA expression values for at least one experimental group.
(CSV)

Table S4 **Spearman correlation coefficients of allinteractions for non-invasive and invasive bladder cancer tissue samples.** Only, interactions were selected that exhibit a negative correlation, i.e. ρ≤−0.4, between the normalized miRNA and mRNA expression values for at least one experimental group.
(CSV)

Table S5 **Functional annotation clustering of selected mRNAs using DAVID v6.7.** Starting with the whole tissue collective (8 normal and 16 tumor tissues) we obtained 2480 miRNA-mRNA interactions with a JI > = 0.4. The corresponding 1459 mRNAs of these interaction pairs were analyzed by DAVID software with standard classification stringency. 28.4% (414 mRNAs) of the analyzed mRNAs could be matched into the Kegg pathways.
(PDF)

Table S6 **Differentially regulated interactions for all samples inside the FGF3R pathway.** Only, interactions were selected that exhibit a negative correlation, i.e. ρ≤−0.4, between the normalized miRNA and mRNA expression values for at least one experimental group. Interactions with a Jaccard-index ≥0.40 are shown. The regulation in bladder cancer tissue samples compared to normal tissue samples is indicated.
(PDF)

Table S7 **Differentially regulated interactions for all samples inside the bladder cancer pathway.** Only, interactions are selected that exhibit a negative correlation, i.e. ρ≤−0.4, between the normalized miRNA and mRNA expression values for at least one experimental group. Interactions with a Jaccard-index ≥0.40 are shown. The regulation in bladder cancer tissue samples compared to normal tissue samples is indicated.
(PDF)

TableS8 **List of the shared interactions between our approach CAPE_RNA and each of the other used prediction methods.** Two different collectives were analysed: the entire bladder cancer set of normal (n = 8) and tumor samples (n = 16) and the collective of invasive (n = 8) and non-invasive (n = 8) bladder cancer tumor samples without normal samples. Each of the 500 best interactions were compared. A) Intersection TaLasso - CAPE RNA; B) Intersection GenMiR++ - CAPE RNA; C) Intersection Spearman correlation - CAPE RNA: D) a Meta analysis approach – CAPE RNA.
(CSV)
