# Supplementary figures and images for: A New Algorithm for Integrated Analysis of miRNA-mRNA Interactions Based on Individual Classification Reveals Insights into Bladder Cancer
Source: PLoS One. 2013 May 24;8(5):e64543. doi: 10.1371/journal.pone.0064543 (PMC3663800; doi:10.1371/journal.pone.0064543)

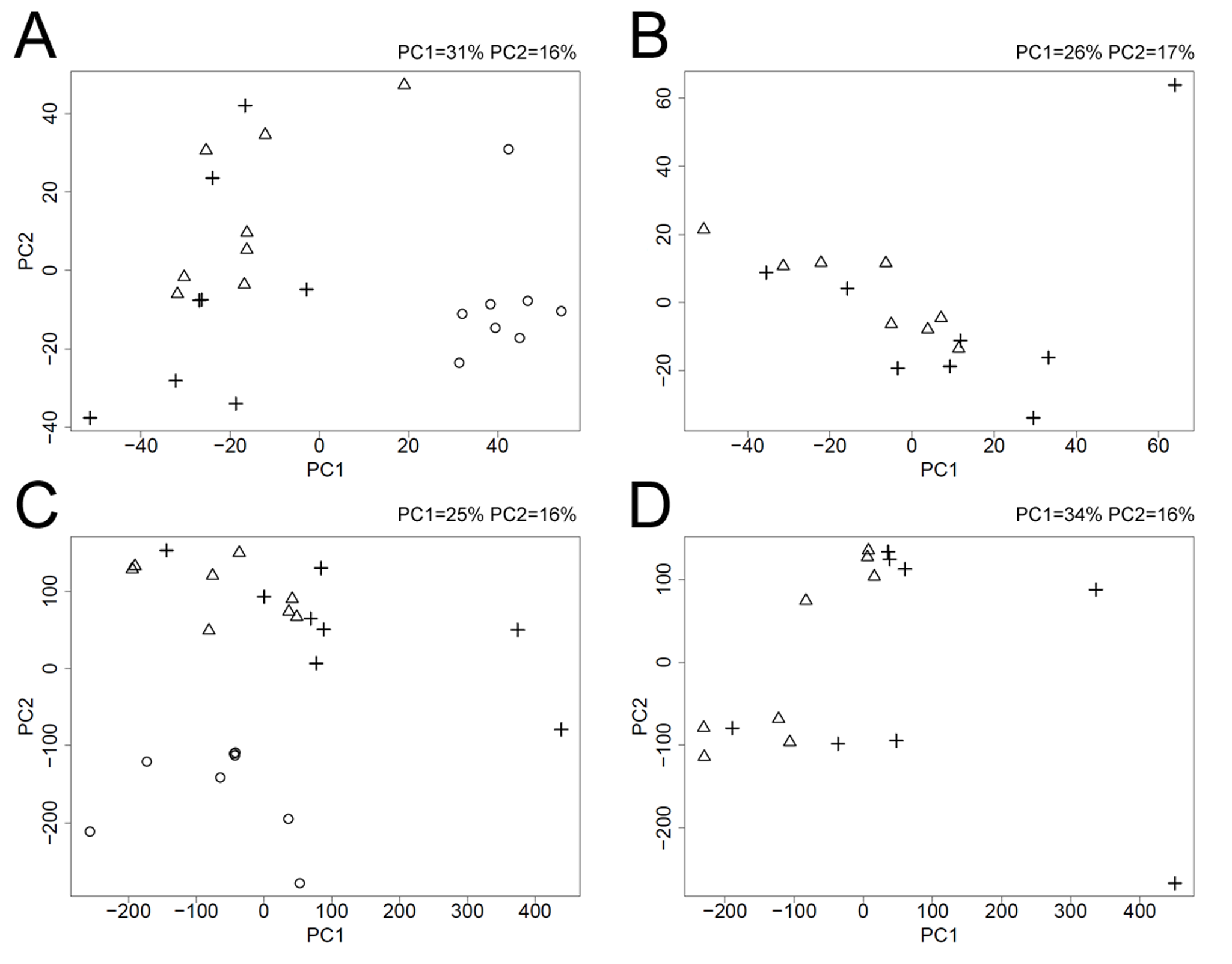

Supplement: Figure S1 — Spread of samples for the bladder cancer data set using normalized expression values. The first two principal components (PC) of the normalized data are shown for the collective of all samples (A and C) and the collective of tumor samples (B and D) using normalized miRNA (A and B) or mRNA (C and D) expression data, respectively. Circles refer to samples of the control group, triangles are samples revealed invasive tumors and crosses refer to samples with non-invasive tumors. (TIF) [file pone.0064543.s001.tif]

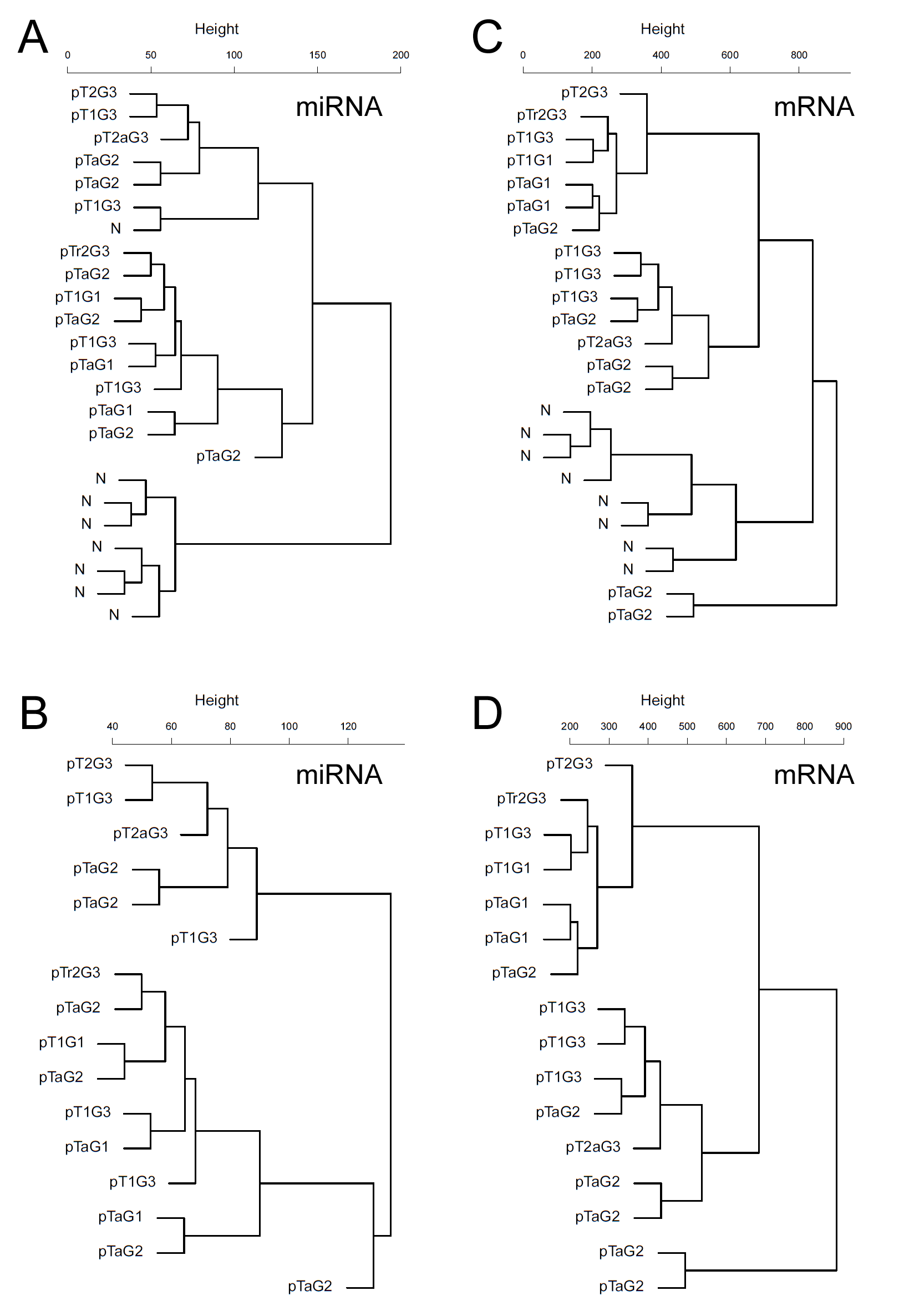

Supplement: Figure S2 — Hierarchical clustering of the bladder cancer data set using normalized expression values. Hierarchical clustering of the normalized expression data was performed using Ward’s method for the collective of all samples (A and C) and the collective of tumor samples (B and D) using normalized miRNA (A and B) or mRNA (C and D) expression data, respectively. (TIF) [file pone.0064543.s002.tif]

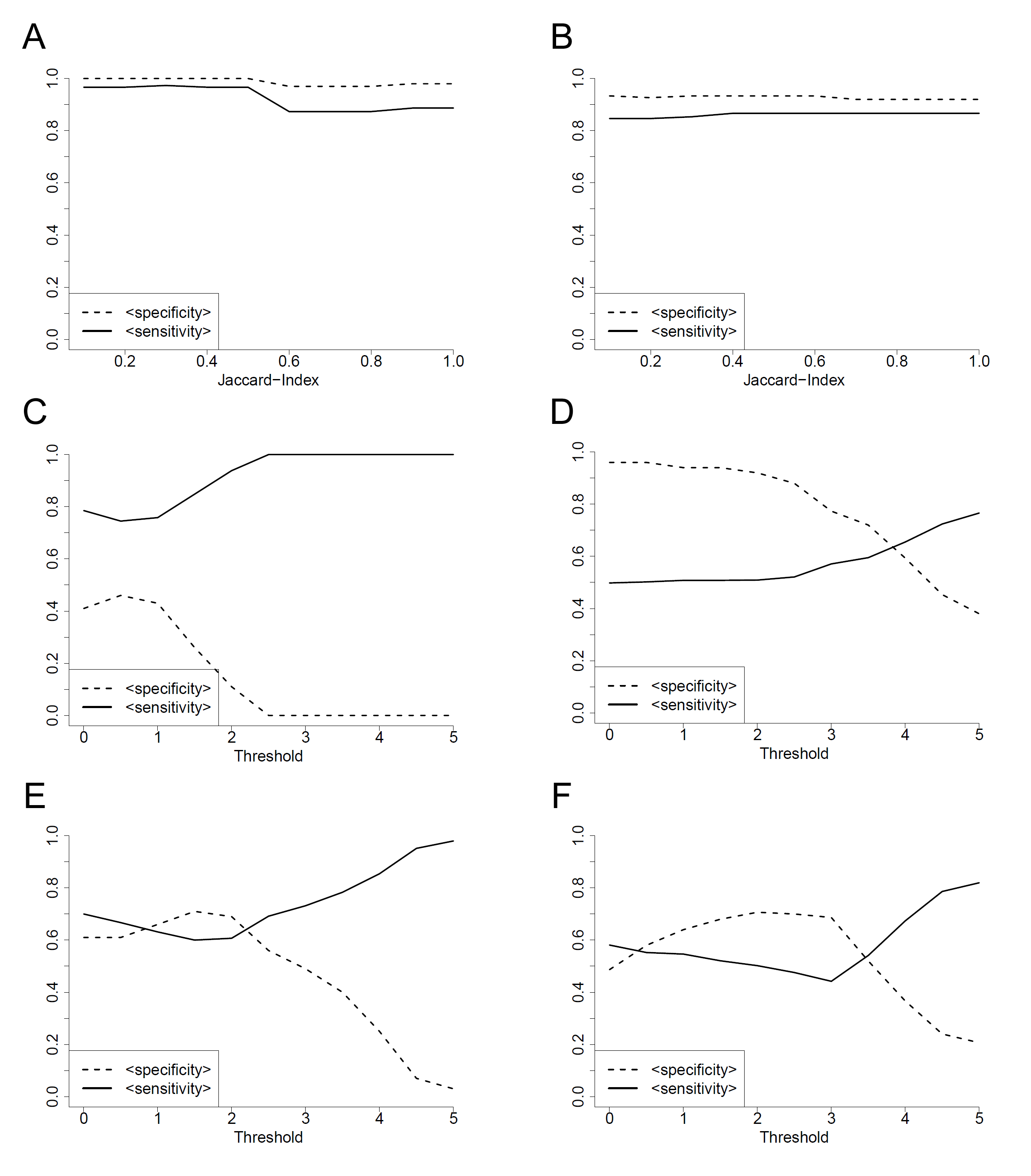

Supplement: Figure S3 — Mean specificities and sensitivities for the colon and prostate cancer data. Models for the colon collective (A) and prostate collective (B) based on our approach, CAPE RNA, were generated from training sets by selecting all interactions with a Jaccard-index equal to or higher than a threshold. Prediction Analysis of Microarrays for R was used to train models for the colon tissue samples based on miRNA (C) and mRNA expression (D), as well as for the prostate collective based on miRNA (E) and mRNA (F) expression. Models were generated with different thresholds. A-F) illustrate the mean specificities and mean sensitivities to classify unknown test sets. (TIF) [file pone.0064543.s003.tif]

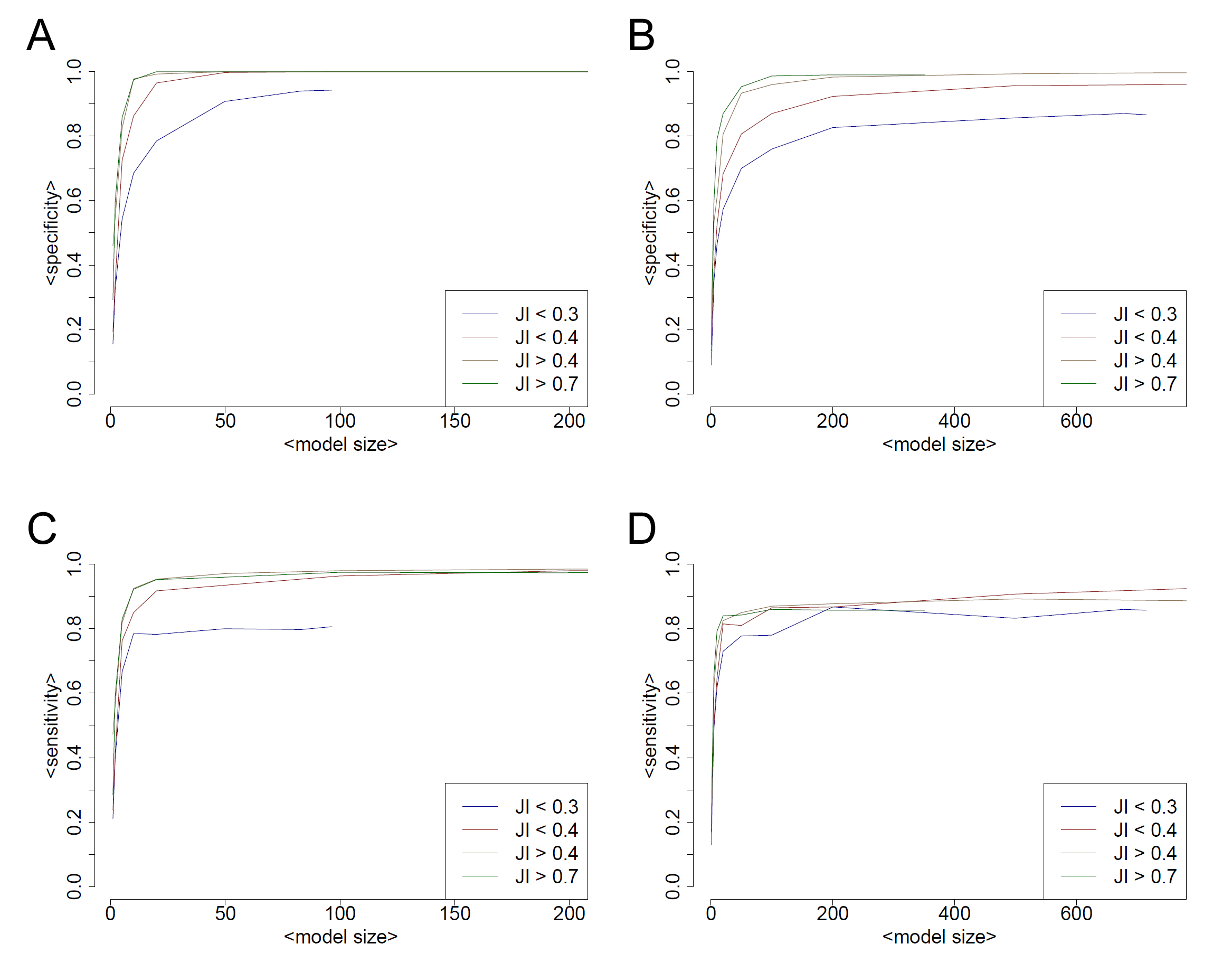

Supplement: Figure S4 — Mean specificities and sensitivities for the bladder cancer data set by randomized models. Models were generated by randomly picking a defined of interactions within a specific range of Jaccard-indexes using our approach, CAPE RNA. The performance of different models was compared to classify unknown test sets: a) specificities and b) sensitivities to discriminate tumor samples from healthy tissue samples, c) specificities and d) sensitivities to discriminate invasive tumor samples from non-invasive tumor samples. (TIF) [file pone.0064543.s004.tif]

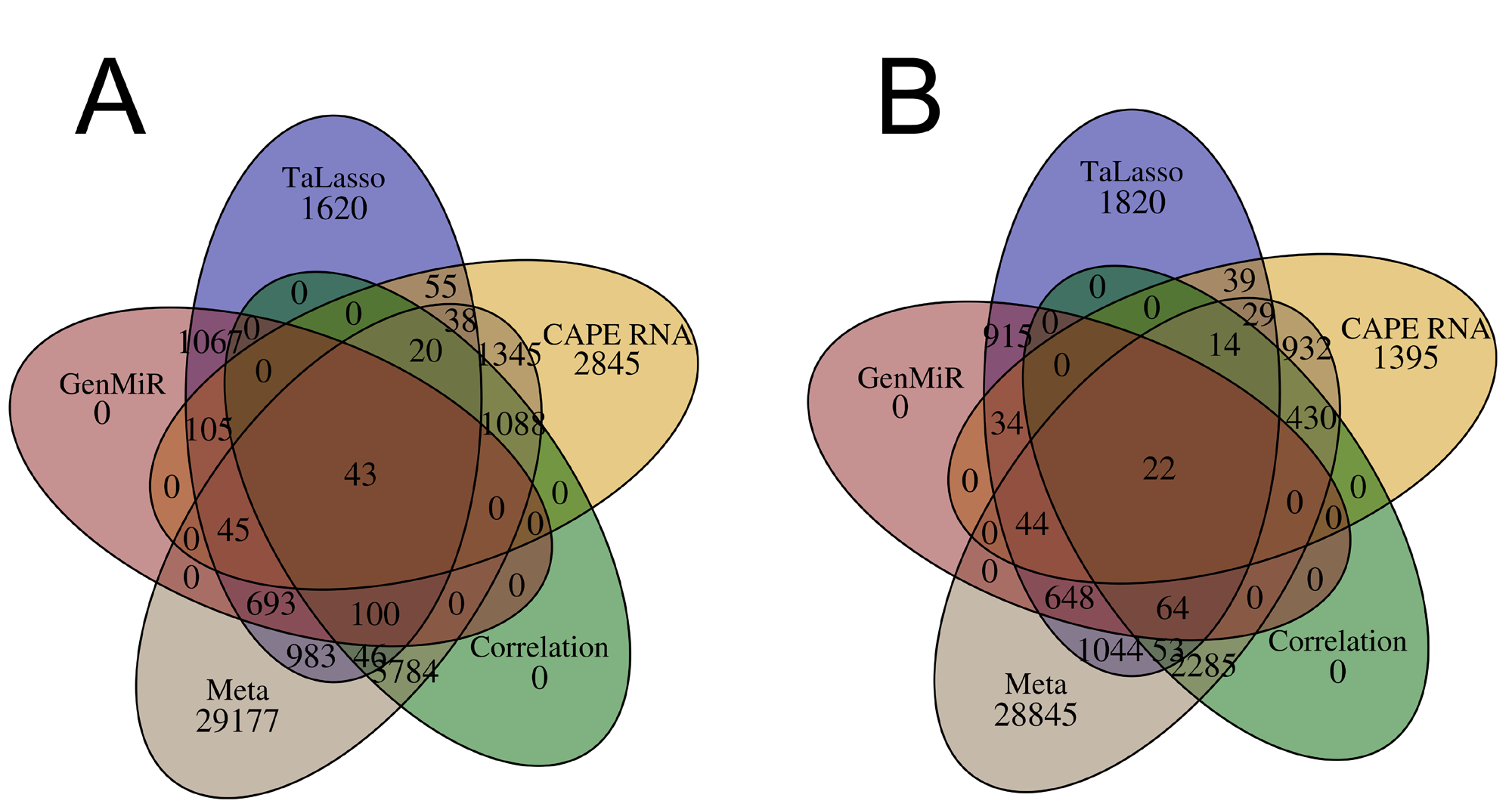

Supplement: Figure S5 — Venn diagram of predicted miRNA-mRNA interactions in bladder cancer derived from five different methods for integrative analysis (TaLasso, GenMiR++, Spearman correlation, a Meta analysis approach and our new algorithm CAPE RNA). Two different collectives were analysed: (A) the entire bladder cancer dataset of normal (n = 8) and tumor samples (n = 16) and (B) only the collective of invasive (n = 8) and non-invasive (n = 8) bladder cancer tumor samples. The number of all predicted miRNA-mRNA interactions by each method were visualized in the Venn diagram. (TIF) [file pone.0064543.s005.tif]
