# Supplementary material for: A New Algorithm for Integrated Analysis of miRNA-mRNA Interactions Based on Individual Classification Reveals Insights into Bladder Cancer
Source: PLoS One. 2013 May 24;8(5):e64543. doi: 10.1371/journal.pone.0064543 (PMC3663800; doi:10.1371/journal.pone.0064543)
Supplement: Table S5 — Functional annotation clustering of selected mRNAs using DAVID v6.7. Starting with the whole tissue collective (8 normal and 16 tumor tissues) we obtained 2480 miRNA-mRNA interactions with a JI > = 0.4. The corresponding 1459 mRNAs of these interaction pairs were analyzed by DAVID software with standard classification stringency. 28.4% (414 mRNAs) of the analyzed mRNAs could be matched into the Kegg pathways. (PDF) [file pone.0064543.s010.pdf]

Table S5: Functional annotation clustering of selected mRNAs using DAVID v6.7. Starting with the whole tissue collective (8 normal and 16 tumor tissues) we obtained 2480 miRNA-mRNA interactions with a JI >= 0.4. The corresponding 1459 mRNAs of these interaction pairs were analyzed by DAVID software with standard classification stringency. 28.4% (414 mRNAs) of the analyzed mRNAs could be matched into the Kegg pathways.

**DAVID Enrichment Score: 3,61**					
**Term**	**Count**	**%**	**Genes**	**PValue**	**Benjamini**
hsa05223: Non-small cell lung cancer	17	1,2	595, 842, 2064, 1869, 5579, 5578, 2309, 5915, 1029, 1871, 1870, 1021, 10000, 4893, 5293, 6789, 6654	0,00000	0,0002
hsa05215: Prostate cancer	21	1,4	595, 842, 5728, 2064, 1869, 596, 2263, 56034, 3479, 1871, 1870, 3480, 9134, 10000, 4893, 5293, 1387, 898, 6654, 6934, 5159	0,00002	0,0006
hsa05214: Glioma	17	1,2	595, 5728, 1869, 5579, 5578, 817, 1029, 3479, 1871, 1870, 3480, 1021, 10000, 4893, 5293, 6654, 5159	0,00003	0,0007
hsa05218: Melanoma	17	1,2	595, 5728, 1869, 56034, 1029, 2252, 3479, 1871, 1870, 3480, 1021, 10000, 4893, 5293, 4286, 4233, 5159	0,00013	0,0022
hsa05212: Pancreatic cancer	16	1,1	595, 842, 2064, 1869, 1029, 2277, 3716, 1871, 1870, 7042, 1021, 10000, 5293, 7422, 4089, 7046	0,00053	0,0061
hsa05220: Chronic myeloid leukemia	16	1,1	861, 595, 1869, 1029, 1871, 1870, 7042, 1021, 10000, 4893, 5293, 6777, 4089, 613, 7046, 6654	0,00083	0,0083
hsa05222: Small cell lung cancer	17	1,2	595, 842, 5728, 1869, 596, 5915, 317, 1286, 1871, 1870, 3685, 9134, 1021, 10000, 5293, 3915, 898	0,00098	0,0087
hsa05219: Bladder cancer	11	0,8	595, 2064, 1869, 4893, 7422, 1029, 2277, 1612, 1871, 2261, 1870	0,00154	0,0110
hsa05213: Endometrial cancer	10	0,7	595, 842, 2064, 5728, 5293, 4893, 10000, 2309, 6654, 6934	0,02251	0,1118
**DAVID Enrichment Score: 2,28**					
**Term**	**Count**	**%**	**Genes**	**PValue**	**Benjamini**
hsa05211: Renal cell carcinoma	16	1,1	3725, 57144, 5908, 5058, 2277, 7042, 2889, 10000, 4893, 5293, 7422, 2549, 1387, 4233, 56924, 6654	0,00038	0,0058
hsa04012: ErbB signaling pathway	17	1,2	2066, 3725, 57144, 2064, 2065, 5579, 5578, 817, 5058, 6198, 10000, 4893, 5293, 2549, 6777, 56924, 6654	0,00146	0,0109
hsa04660: T cell receptor signaling pathway	12	0,8	3725, 57144, 5293, 4893, 10000, 2534, 5058, 2353, 56924, 10451, 6654, 1739	0,25944	0,6190
**DAVID Enrichment Score: 2,01**					
**Term**	**Count**	**%**	**Genes**	**PValue**	**Benjamini**
hsa04270: Vascular smooth muscle contraction	19	1,3	115, 112, 5579, 5578, 3708, 800, 775, 5332, 10672, 5613, 1909, 3778, 4638, 5500, 107, 4629, 5592, 9475, 5581	0,00363	0,0236
hsa04540:	16	1,1	115, 112, 5579, 5578, 3708, 5332, 56034,	0,00486	0,0290

Gap junction			1902, 5613, 3356, 3358, 4893, 107, 5592, 6654, 5159		
hsa04912: GnRH signaling pathway	14	1,0	3725, 115, 112, 5579, 5578, 817, 4215, 3708, 5332, 775, 5613, 4893, 107, 6654	0,05217	0,1831
**DAVID Enrichment Score: 1,63**					
**Term**	**Count**	**%**	**Genes**	**PValue**	**Benjamini**
hsa05414: Dilated cardiomyopathy	18	1,2	6444, 1756, 782, 783, 6443, 115, 112, 775, 3479, 5613, 488, 7042, 3685, 7168, 3696, 107, 70, 7170	0,00099	0,0083
hsa05410: Hypertrophic cardiomyopathy	15	1,0	1756, 6444, 782, 783, 6443, 775, 3479, 488, 7042, 3685, 7168, 5565, 3696, 70, 7170	0,00789	0,0418
hsa05412: Arrhythmogenic right ventricular cardiomyopathy	11	0,8	6444, 1756, 782, 3685, 783, 6443, 775, 3696, 1824, 488, 6934	0,08576	0,2701
hsa04260: Cardiac muscle contraction	8	0,5	782, 783, 7168, 775, 70, 477, 7170, 488	0,44990	0,7613
**DAVID Enrichment Score: 0,40**					
**Term**	**Count**	**%**	**Genes**	**PValue**	**Benjamini**
hsa04666: Fc gamma R-mediated phagocytosis	12	0,8	3984, 6198, 5293, 10000, 4651, 5579, 5578, 10810, 5058, 10451, 65108, 5581	0,14586	0,4179
hsa04660: T cell receptor signaling pathway	12	0,8	3725, 57144, 5293, 4893, 10000, 2534, 5058, 2353, 56924, 10451, 6654, 1739	0,25944	0,6190
hsa04664: Fc epsilon RI signaling pathway	9	0,6	5293, 4893, 10000, 5579, 5578, 2534, 10451, 6654, 5581	0,29820	0,6568
hsa04662: B cell receptor signaling pathway	8	0,5	3725, 5293, 4893, 10000, 5579, 2353, 10451, 6654	0,40893	0,7279
hsa04650: Natural killer cell mediated cytotoxicity	8	0,5	5293, 4893, 5579, 5578, 2534, 5058, 10451, 6654	0,92445	0,9893
hsa04620: Toll-like receptor signaling pathway	4	0,3	3725, 5293, 10000, 2353	0,99098	0,9995
