# Supplementary material for: A New Algorithm for Integrated Analysis of miRNA-mRNA Interactions Based on Individual Classification Reveals Insights into Bladder Cancer
Source: PLoS One. 2013 May 24;8(5):e64543. doi: 10.1371/journal.pone.0064543 (PMC3663800; doi:10.1371/journal.pone.0064543)
Supplement: Table S6 — Differentially regulated interactions for all samples inside the FGF3R pathway. Only, interactions were selected that exhibit a negative correlation, i.e. ρ≤−0.4, between the normalized miRNA and mRNA expression values for at least one experimental group. Interactions with a Jaccard-index ≥0.40 are shown. The regulation in bladder cancer tissue samples compared to normal tissue samples is indicated. (PDF) [file pone.0064543.s011.pdf]

Table S6: Differentially regulated interactions for all samples inside the FGF3R pathway. Only, interactions were selected that exhibit a negative correlation, i.e. ρ ≤ -0.4, between the normalized miRNA and mRNA expression values for at least one experimental group. Interactions with a Jaccard-index ≥ 0.40 are shown. The regulation in bladder cancer tissue samples compared to normal tissue samples is indicated.

miRNA	miRNA expression	Gene Symbol	geneID	Gene expression	Jaccard -index
miR-200c	Up	PRKCA	5578	Down	0.69
miR-204	Down	SOS1	6654	Up	0.63
miR-494	Up	PRKCB	5579	Down	0.57
miR-30e	Up	PDGFRB	5159	Down	0.54
miR-100	Down	FGFR3	2261	Up	0.53
miR-26a	Down	DAPK1	1612	Up	0.51
miR-27b	Down	SOS1	6654	Up	0.51
miR-29b	Up	PDGFRB	5159	Down	0.46
miR-152	Down	SOS1	6654	Up	0.44
miR-340	Down	DAPK1	1612	Up	0.43
miR-182	Up	PRKCE	5581	Down	0.43
miR-132	Down	SOS1	6654	Up	0.42
miR-200a	Up	PRKCE	5581	Down	0.40
