# Supplementary material for: A New Algorithm for Integrated Analysis of miRNA-mRNA Interactions Based on Individual Classification Reveals Insights into Bladder Cancer
Source: PLoS One. 2013 May 24;8(5):e64543. doi: 10.1371/journal.pone.0064543 (PMC3663800; doi:10.1371/journal.pone.0064543)
Supplement: Table S7 — Differentially regulated interactions for all samples inside the bladder cancer pathway. Only, interactions are selected that exhibit a negative correlation, i.e. ρ≤−0.4, between the normalized miRNA and mRNA expression values for at least one experimental group. Interactions with a Jaccard-index ≥0.40 are shown. The regulation in bladder cancer tissue samples compared to normal tissue samples is indicated. (PDF) [file pone.0064543.s012.pdf]

Table S7: Differentially regulated interactions for all samples inside the bladder cancer pathway. Only, interactions are selected that exhibit a negative correlation, i.e. $\rho \leq -0.4$, between the normalized miRNA and mRNA expression values for at least one experimental group. Interactions with a Jaccard-index ≥ 0.40 are shown. The regulation in bladder cancer tissue samples compared to normal tissue samples is indicated.

miRNA	miRNA expression	Gene Symbol	geneID	Gene expression	Jaccard -index
miR-125b	Down	ERBB2	2064	Up	0.74
miR-30a	Down	E2F3	1871	Up	0.57
miR-100	Down	FGFR3	2261	Up	0.53
miR-26a	Down	DAPK1	1612	Up	0.51
miR-28-5p	Down	NRAS	4893	Up	0.49
miR-497	Down	CCND1	595	Up	0.49
miR-495	Down	E2F2	1870	Up	0.49
miR-182	Up	FIGF	2277	Down	0.49
miR-497	Down	E2F3	1871	Up	0.48
miR-125b	Down	CDKN2A	1029	Up	0.46
miR-195	Down	E2F3	1871	Up	0.45
miR-136	Down	E2F1	1869	Up	0.44
miR-320a	Down	E2F3	1871	Up	0.44
miR-340	Down	DAPK1	1612	Up	0.43
miR-29a	Down	VEGFA	7422	Up	0.43
miR-152	Down	E2F3	1871	Up	0.41
